# Contributions to Estimating the Water-Holding Capacity in Fresh Pork Hams Using NMR Relaxometry

**DOI:** 10.3390/foods14132329

**Published:** 2025-06-30

**Authors:** Víctor Remiro, María Isabel Cambero, María Dolores Romero-de-Ávila, David Castejón, José Segura, María Encarnación Fernández-Valle

**Affiliations:** 1 Food Technology Department, Faculty of Veterinary Medicine, Complutense University of Madrid, Av. Puerta de Hierro s/n, 28040 Madrid, Spain; vremiro@ucm.es (V.R.); icambero@ucm.es (M.I.C.); mdavilah@ucm.es (M.D.R.-d.-Á.); josesegu@ucm.es (J.S.); 2 ICTS Complutense Bioimaging (BioImac), Complutense University of Madrid, Paseo Juan XXIII 1, 28040 Madrid, Spain; dcastejon@ucm.es

**Keywords:** meat quality, WHC, physicochemical meat properties, MRI, TD-NMR, T1 and T2 relaxation times

## Abstract

Determining the technological quality of fresh meat pieces is essential in the meat industry to ensure the production of high-quality products. For this purpose, nuclear magnetic resonance (NMR) is a non-destructive and non-invasive technique that appears as an alternative to traditional methodologies. The objective of this work is to determine the potential of magnetic resonance imaging (MRI) and time-domain (TD-NMR) relaxometry for determining the physicochemical characterization of fresh hams with different industrial destinations (both fresh and cured products, such as dry-cured ham). For this study, the *biceps femoris*, *semimembranosus,* and *semitendinosus* muscles of 20 fresh hind legs from white pigs, classified into four categories according to their fat content, were analyzed. The *semitendinosus* muscle was selected as a model, and positive and negative correlations were obtained between different physicochemical parameters and the longitudinal (T1) and transverse (T2) relaxation times obtained by MRI and TD-NMR. Regression models using T1 and T2 were also developed to predict the muscle water-holding capacity (WHC) and drip loss, using high, medium, and low magnetic field NMR (R^2^ > 0.80). Therefore, MRI and TD-NMR could be considered as highly suitable and accurate non-destructive techniques for the WHC determination in the meat industry.

## 1. Introduction

Water-holding capacity (WHC) refers to the ability of meat to retain intrinsic and added water during various stages of handling, including cutting, grinding, pressing, storage, and thermal processing. WHC, along with other parameters such as pH, color, or intramuscular fat content (IMF), is directly related to the organoleptic quality of meat and is also a critical attribute to consider when determining its industrial application or the type of processing it will undergo. Meat juiciness, perceived as moisture during mastication, depends directly on WHC, along with IMF. WHC is also indirectly related to the perception of flavor and tenderness [1]. Moisture losses, commonly called drip, purge, or cook loss, are inversely proportional to WHC. It has been reported that up to 50% or more of pork production exhibits unacceptably high levels of purge or drip loss [2]. In processed products, retained water post-cooking is expressed as cook yield, which is directly related to WHC [3].

Although WHC is a crucial criterion of quality assessment in meat processing, no precise analytical method for its measuring has been established. The variety of evaluation methods complicates comparisons and unbiased assessments. There are more than 100 methodologies used to obtain WHC [4]. Direct methods are based on weight losses related to water loss due to the application of centrifugation, compression forces, or cooking. Indirect methods can be used through techniques based on spectroscopy, light scattering, electrical conductivity, microwaves, or nuclear magnetic resonance (NMR) [5].

NMR is a spectroscopic technique that analyzes the interaction between matter and electromagnetic radiation through the intrinsic magnetic properties of atomic nuclei [6,7]. NMR offers different alternatives such as magnetic resonance imaging (MRI) and time-domain relaxometry (TD-NMR). These methodologies provide macrostructural, microstructural, and compositional information for the sample through longitudinal (T1) and transverse (T2) relaxation times [8,9]. Both T1 and T2 are related to food structure, water distribution, and mobility [10,11], identifying NMR as an alternative to traditional methodologies for food analysis. NMR is a non-destructive or minimally invasive technology, requires minimal sample preparation, and enables rapid, simultaneous quantification of multiple components. Moreover, NMR provides detailed molecular-level information, enhancing the understanding of food structure and composition. These features make it particularly valuable for quality control and authenticity assessment in the food industry. As a well-established technique, there are numerous investigations of the application of NMR for the determination of meat quality, such as water distribution, fat content, and authentication. However, there are a number of limitations, such as the cost of equipment, the standardization of analysis and results, and the possibility of performing real-time and on-line studies, which are essential for its application at the industrial level. This possible application in industry would require the development of reliable models relating resonance parameters to meat quality. While models for specific quality traits are highly accurate, comprehensive models covering all aspects of meat quality are still under development [12].

T2 can be divided into several signals associated with protons and the mobility of different types of muscle water: (1) water bound to proteins, which exhibits low mobility and corresponds to the lowest T2 (T2_b_); (2) immobilized water, which is located inside the muscle but is not bound to proteins and corresponds to intermediate T2 values (T2_1_); and (3) free water, which corresponds to the longest T2 (T2_2_) [2,13,14].

In fact, regarding the different types of water in meat matrices, most studies are focused on the T2 relaxation time. Correlations between the WHC obtained by direct methods and the relaxation times T2_1_ and T2_2_ have been established in pork *longissimus dorsi* (LD) and *semitendinosus* (ST) [14]. A decrease in T2 relaxation times associated with immobilized water and free water protons related to a higher WHC was observed in beef [15]. WHC was also estimated through T2 in turkey breast [16].

As stated, when estimating WHC, most studies are focused on the T2 relaxation time, without taking into account T1. Because T1 depends on water mobility, fat content, and protein structure, T1 values can also provide complementary information regarding fat and water protons [17]. The information provided by both parameters allows for the assessment of the degree of water interaction with various structures within the muscle tissue and consequently, the water-binding or retention capacity of the matrix.

This work aims to take a step toward the use of MRI and TD-NMR, of either low or high magnetic field strength, for the physicochemical and structural characterization of fresh hams with different levels of fattening and with different industrial destinations by establishing correlations between and regression models for the NMR parameters (T1 and T2) and physicochemical parameters crucial to ensure the quality of the final product.

## 2. Materials and Methods

### 2.1. Meat Sampling

For this study, right and left hind legs (fresh hams) from 20 white pigs [Duroc × (Landrace × Large white)], supplied by a meat company 48 h after slaughter, were used. The right leg of each animal was used to estimate the total fat content, while the left leg was used to obtain the *biceps femoris* (BF), *semimembranosus* (SM), and *semitendinosus* (ST) muscles and their subsequent physicochemical characterizations. The different determinations were carried out on the same day that the pieces were obtained.

### 2.2. Determination of Fat Content, pH, and Weight of Fresh Hams

All the fresh hams were weighed, and their pH was determined in the SM muscle 24 h after slaughter by puncture with a Crison pH25+ pH meter (Hach, Loveland, CO, USA). The total fat content of all the pieces was determined using an X-ray scanner (Multiscan Technologies, Cocentaina, Alicante, Spain). Additionally, the right fresh ham of each animal was deboned after ablation of the distal portion at the tarsus. The resulting material from this whole leg was used to obtain a homogenate, after mincing and mixing, and was used to corroborate the fat content using the Soxhlet extraction method [18]. The fat content, determined using the X-ray equipment, was used to classify the left fresh ham from the same animal into four categories (lean, medium fat content, fatty, and very fatty). Five pieces from each category were selected for the study.

### 2.3. Physicochemical Characterization of Different Muscles

The following determinations were carried out in the BF, SM, and ST muscles of left fresh hams: The ash content (%) was determined gravimetrically after incinerating the samples at 500 °C for 12 h. Dry matter (DM, %) and water content (WC, %) were measured gravimetrically by drying the samples at 110 °C until they reached a constant weight [19]. Water-holding capacity (WHC, %) was assessed by compression, following the method described by Kauffman et al. [20]. Drip loss (%) was calculated as the percentage of weight loss of the sample after storage at 4 °C in air for 24 h. Protein content (%) was determined using the Kjeldahl method [21], while intramuscular fat content (IMF, %) was analyzed using the Soxhlet extraction method [18]. The pH was measured by direct puncture with a Crison pH25+ meter (Hach, Loveland, CO, USA). The color parameters were evaluated using the CIELAB system, specifically L* (lightness), a* (red-green), and b* (yellow-blue), measured with a Minolta CR-300 tristimulus colorimeter (Konica Minolta, Ramsey, NJ, USA). Each analysis was conducted in triplicate.

### 2.4. Nuclear Magnetic Resonance Analysis

#### 2.4.1. Magnetic Resonance Imaging (MRI)

##### High Magnetic Field MRI (4.7 T)

The MRI analysis was carried out on the ST muscle using a 4.7 T Bruker Biospec 47/40 system (Bruker BioSpin, Ettlingen, Germany), equipped with a 12 cm gradient coil and a 7 cm volume RF coil. All measurements were performed at a constant temperature of 18 °C. T2 relaxation times were determined using a multi-echo Carr–Purcell–Meiboom–Gill (CPMG) spin-echo sequence. Sixty echoes were recorded, with echo times (TE) ranging from 8.3 to 498 ms, ensuring the coverage of a broad spectrum of T2 values and capturing the full decay of the signal. The repetition time (TR) was set at 6493.9 ms. Additional parameters included a field of view (FOV) of 9 × 4.5 cm^2^, a slice thickness of 1.5 mm, three slices per scan, and a matrix size of 256 × 128. For T1 measurements, a spin-echo sequence was employed. Six images were acquired, with logarithmically spaced TR values between 79.5 and 6079.5 ms, while TE was fixed at 9 ms. The geometric parameters matched those used in the T2 protocol.

##### Medium Magnetic Field MRI (1 T)

The MRI was conducted on the ST muscle using a 1 T Bruker ICON-1T spectrometer (Bruker GmbH, Ettlingen, Germany), operating at a frequency of 42.58 MHz and equipped with actively shielded gradients capable of reaching amplitudes of up to 450 mT/m in all spatial directions. Relaxation parameters (T1 and T2) were quantified using spin-echo sequences. The imaging parameters were as follows: a FOV of 80 × 40 mm^2^, a slice thickness of 1.5 mm, and three coronal slices per acquisition, maintaining a constant in-plane resolution of 350 μm^2^. For T2 measurements, a series of 50 echoes was acquired, with TE ranging linearly from 6 to 300 ms, with a fixed TR of 5000 ms. To determine T1 values, eight experiments were acquired using variables TR (50, 75, 150, 300, 600, 1000, 3000, and 6000 ms), with a TE fixed at 7 ms.

##### Relaxation Time Calculation from MR Image Series

The T1 and T2 values from the MR images were calculated using our own scripts developed with MATLAB R2024b (The MathWorks Inc., Natick, MA, USA). First, a single region of interest (ROI) on the central slice of the three acquired samples was defined. This ROI covered most of the area of the sample, but the borders of the image of the sample were excluded to avoid possible areas of inhomogeneity of the signal. Then the curve of the mean signal of this ROI in every image of the T1 or T2 series was fitted to the corresponding function. For T1, the recovery curve was modeled using the function $S(TR)=C+S_{0}\;(1-e^{-\frac{TR}{T1}})$, where C corresponds to the offset of the minimum signal; S(TR) is the signal at each recovery time TR; and S_0_ is the signal at full recovery. For T2 calculation, the signal decay was fitted to the function $S\left(TE\right)=C+S_{0}e^{\frac{-TE}{T2}},$ where S(TE) is the signal value at each echo time TE, and S_0_ represents the theoretical signal intensity at TE = 0.

#### 2.4.2. Time-Domain Nuclear Magnetic Resonance (TD-NMR)

The TD-NMR analysis was performed on the ST muscle using a Bruker Minispec LF90II analyzer (Bruker BioSpin, Ettlingen, Germany), operating at a magnetic field strength of 0.15 T, equipped with a 9 cm RF probe. All measurements were carried out at 36 °C. T1 values were obtained using an inversion recovery pulse sequence, with variable inversion times (TI) ranging from 5 to 2000 ms. A total of 12 TI values were acquired, with a TR of 2 s, and signal averaging was performed over four scans per TI. For T2 quantification, a CPMG multi-echo sequence was applied. The TE was set to 403.52 μs, and 4096 echoes were collected. The TR was maintained at 2 s, and each measurement was averaged over 16 scans to improve the signal-to-noise ratio. Relaxation times were analyzed using custom-built software developed with MATLAB R2024b (The MathWorks Inc., Natick, MA, USA). Initial estimation of average T1 and T2 values for each sample was achieved by fitting the signal curves to monoexponential models: $S(TI)=C+S_{0}(1-2e^{\frac{-TI}{T1}}),$ for T1 calculation, and $S(TE)=C+S_{0}e^{\frac{-TE}{T2}},$ for T2. Subsequently, continuous distributions of T1 and T2 were computed by applying an inverse Laplace transform, following the algorithm proposed by Bjarnason & Mitchell [22].

### 2.5. Statistical Analysis

All statistical analyses were performed using Statgraphics 19-X64 for Windows (Statistical Graphics Corp., Rockville, MD, USA). After verifying the normal distribution of the data (at a 90% confidence level) using the Shapiro–Wilk test, simple and multifactorial ANOVA with physicochemical parameters were performed. A simple ANOVA of the parameters obtained by NMR for the ST muscle was also performed. Results were expressed as mean values ± standard deviations. Fisher’s least significant difference (LSD) procedure at a 95% confidence interval was used to discriminate between means. *F*-value and *p*-value were considered. To evaluate the correlations between the NMR parameters and the physicochemical properties, Pearson’s correlation coefficient (r) was calculated. Statistical significance was assessed using the *p*-value at a 95% confidence level (*p* < 0.05).

Simple and multiple regression models were developed to further explore the relationships between WHC and drip loss and the NMR parameters. Model performance was evaluated using the Durbin–Watson test to assess autocorrelation, with a significance threshold set at 95% confidence. Key metrics such as the coefficient of determination (R^2^), standard error (SE), root mean square error (RMSE), *F*-value, and *p*-value were considered. The interaction between WHC and drip loss with NMR T1 and T2 relaxation times was studied using surface response models. These models followed the following equation: $Y=\beta_{0}+\beta_{1}T1+\beta_{2}T2+\beta_{11}{T1}^{2}+\beta_{22}{T2}^{2}+\beta_{12}T1T2$, where *Y* represents the predicted WHC or drip loss. The coefficients β_0_ through β_12_ represent the intercept, linear, quadratic, and interaction effects. A backward stepwise selection method was applied to identify significant predictors in the multivariate models [23]. The statistical significance of equation coefficients was evaluated using the F-test for each response variable.

## 3. Results and Discussion

### 3.1. Categorization and Characterization of Fresh Hams

The fat content of fresh hams is considered a fundamental aspect for selecting the commercial destination of these meat pieces and for the sensory quality of the final product. For this reason, one of the main criteria commonly used for the categorization of fresh hams in the meat industry is fat content, distinguishing between lean, semi-fatty, and fatty fresh hams [24]. The 20 fresh pork legs analyzed in this study were categorized according to total fat content into four categories, from lean (category A) to fatty (category D), in addition to semi-fatty fresh hams (categories B and C) (Table S1). Two other parameters that were considered when selecting the meat pieces for this study were the pH of the fresh hams 24 h post-mortem and the total weight of the hams. Fresh hams with pH values between 5.5 and 6.0 and a weight between 12 and 13 kg were selected (Table S1). In this way, the matrices used were within the ranges of normality in the meat industry for obtaining, among other products, prime dry-cured hams [25].

### 3.2. Physicochemical Analysis of the Different Muscles of the Fresh Hams

The *Biceps femoris* (BF), *semimembranosus* (SM), and *semitendinosus* (ST) are the most representative muscles of fresh and dry-cured ham, and they are characterized by their different composition and by the different distribution of IMF in each [26]. BF and ST are considered internal muscles, while SM is an external muscle [27] (Figure S1). In this study, a complete physicochemical characterization of each of these muscles was carried out on fresh hams of the four previously mentioned fat categories (Table 1A–C). In addition, Table 2 presents a multifactorial ANOVA showing the influence of muscle, fat category, and their interaction.

The SM muscle presented the lowest DM and IMF values and the highest ash and protein content in all fat categories. However, the ST presented an inverse behavior, with the highest values of DM and IMF content and the lowest protein content (Table 1A). The results obtained in the present study followed the same trend as the results obtained by Kim et al. [28] in their study of 21 fresh pork muscles, as well as by García-García et al. [9] in the fresh hams with which they began their study of dry-cured ham manufacturing. From the multifactorial analysis (Table 2), all parameters were affected by muscle type and fat category. It should be noted that IMF was the component most affected by muscle type (higher *F*-value), due to the different distribution of fat in the ham muscles [29]. The effect of the interaction between both variables was also observed, except for ash and protein content (Table 2).

Lower pH values for the SM muscle were detected in the lean (A) and fat (D) categories than those obtained for BF and ST. These results were associated with a greater presence of type IIB fibers in those muscles than in ST, which are glycolytic fibers showing fast contraction, lower myoglobin content, low lipid content, and high glycogenic content [30]. In spite of this, all pH values obtained were normal values in these types of muscles (Table 1B) [9]. pH was influenced by both muscle type and fat category, but not by the interaction of the two (Table 2).

As for the color parameters, in the categories with the highest fat content in the pork leg (C and D), the ST muscle presented higher values of L* due to the higher luminosity resulting from the increased amount of IMF [31]. The same result occurred for the parameter a* and b* in all categories, with the exception of category D for a*, where the values of this parameter were equal in the three muscles (Table 1C). The redder color of the ST muscle (higher a* values) compared to that of the BF and SM muscles was associated with the higher myoglobin content in type I fibers, which are those found in greater quantities in ST [32]. Realini et al. [33] obtained similar results to those obtained in this study, associating paler shades to muscles with a predominance of IIB fibers (SM and BF). All three color coordinates were affected by muscle type (especially a* and b*), while L* and a* were also influenced by the fat category. As for the interaction between the two variables, only the L* and b* coordinates were affected (Table 2).

As for the parameters related to WC, although there was a significant influence of muscle type on WHC, the main differences were observed according to the fat category (Table 2). Muscles corresponding to the fattier categories (C and D) showed a tendency toward a lower WHC compared to those for the lean categories (A and B). Some authors observed no relationship between WHC and fat content in white pork LD muscle [34], which may be attributed to the smaller range of IMF variability in their samples compared to those analyzed in the present study. Nevertheless, a similar inverse relationship to the one observed in our results was also reported by Daszkiewicz et al. [35] in white pork LD muscle. Regarding drip loss, and inversely to the WHC, muscles of the lean categories showed a tendency to lower values compared to those of the fat categories (Table 1B). This parameter was statistically influenced by both fat category and muscle type, but showed no influence of the interaction between the two (Table 2).

In summary, the multifactorial analysis confirmed that both intrinsic muscle characteristics and fat content play a critical role in determining the technological quality of fresh hams.

### 3.3. MRI and TD-NMR Analysis

Following the characterization of the muscles, the ST muscle was selected for NMR analysis, as well as for the development of regression models and the establishment of correlations with physicochemical parameters.

The ST muscle was chosen for further study based on (i) the characteristic morphology of this muscle, with a very similar IMF distribution in all fresh hams [36,37]; (ii) the fact that this muscle presented the highest IMF content, regardless of the fat category of the fresh hams (Table 1A); (iii) its low intragroup variability in IMF content (when comparing fresh hams within the same fat level), with coefficients of variability between 4% and 5%, whereas in the BF and SM muscles, the IMF content variability ranged from 2–10% and 4–15%, respectively; (iv) the fact that its IMF content enabled the differentiation between lean from fatty or very fatty legs (Table 1A).

**Figure 1:**
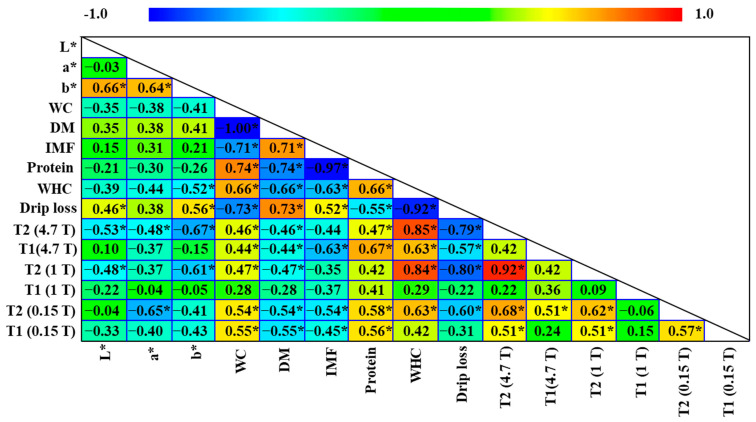
Pearson correlation coefficients obtained between different physicochemical parameters and longitudinal (T1) and transverse (T2) relaxation times (ms) obtained by magnetic resonance imaging (1 and 4.7 T) and time-domain relaxometry (0.15 T) of *semitendinosus* muscle. WC: water content; DM: dry matter; IMF: intramuscular fat content; WHC: water-holding capacity. * *p*-values < 0.05 indicate statistical significance of Pearson’s correlation.

Values of T1 and T2 for ST muscle were obtained using MRI and TD-NMR (Table 3). Higher T1 values were observed with an increasing magnetic field, as described by other authors [38]. T1 ranged between 306 and 370 ms at 0.15 T, depending on the fat category of the hams. However, at both low and high magnetic field strengths, T1 did not allow for the differentiation of fresh hams from different fat categories. At 1 T, T1 values ranged between approximately 710 and 770 ms, while at 4.7, T ranged between 1210 and 1320 ms. In contrast to T1, the effect of magnetic field strength is not as evident at T2, as indicated in the literature [38]. T2 values ranging between 40 and 57 ms were observed for all three magnetic fields. Only T2 values obtained at 0.15 T differed significantly (*p* < 0.05) according to fat category. These results may be attributed to the interaction between the relaxation times of water and fat protons. Considering the inverse relationship between WC and IMF (Figure 1), the relaxation times of fat are likely to have a greater influence in high-fat categories, where WC is lower, and conversely, water relaxation times dominate in leaner categories. The results obtained suggest that fat accumulation may be more evident in low magnetic fields, where the relaxation times of water and fat protons are more closely aligned.

Figure 1 shows the Pearson correlation coefficients of different physicochemical parameters and NMR monoexponential relaxation times obtained at high, medium, and low magnetic fields (4.7, 1 and 0.15 T, respectively) in the ST muscle. The expected results were observed, such as an inverse correlation between protein and fat content. A positive correlation was observed between protein content and T1 and T2. This is due to the fact that the increase in protein content and the decrease in fat content is associated with an increase in the amount of water in the muscle. Water displays a higher T1 and T2 than does fat, which increases the value of these parameters [39]. As DM increases, WC and T2 decrease, establishing an inverse relationship. Directly proportional behavior between T2 and WC has been previously described in salting and maturation processes for pork loin [13] and dry-cured ham [11].

As widely accepted, the WHC showed a negative correlation with drip loss (Figure 1). This same trend is observed in the relationship with T2 and T1 relaxation times obtained with high, medium, and low field spectrometers, which was positive for WHC and negative for drip loss. The highest correlations for both WHC and drip loss were obtained with T2. The lower correlation observed at 0.15 T compared to 1 T and 4.7 T could be explained by the greater influence of the fat category at 0.15 T, observed previously at both T1 and T2 (Table 3). Regarding the relationship of aqueous parameters with CIELAB color coordinates, a negative correlation was observed between L* and WHC, along with a positive correlation with drip loss. This behavior is attributed to the fact that muscles with low WHC tend to release more water, which remains on the surface of the tissue and generates greater light scattering, thus increasing the lightness (L*) of the muscle [40]. This inverse relationship with WHC was also reflected with an inverse correlation between L* and T2.

The WHC is a key parameter of fresh meat quality and has a direct impact on the quality of the final product [41]. For this reason, regression models were developed to study its relationship with NMR relaxation times. Firstly, following the highest value of the Pearson correlation coefficient (Figure 1), linear regression models were established between the T2 obtained at different magnetic fields and WHC (Table S2). The three models obtained at high (4.7 T), medium (1 T), and low magnetic fields (0.15 T) were statistically significant (*p* < 0.05) (R^2^ = 0.70; 0.70; 0.36 for 4.7 T, 1 T and 0.15 T, respectively). The lower R^2^ observed at 0.15 T than at 1 and 4.7 T may be related to the great influence of the fat category on T2 observed at a low magnetic field (Table 3).

The possibility of obtaining regression models that allow WHC to be predicted through T2 has also been studied by other authors in similar meat matrices [15,42].

**Figure 2:**
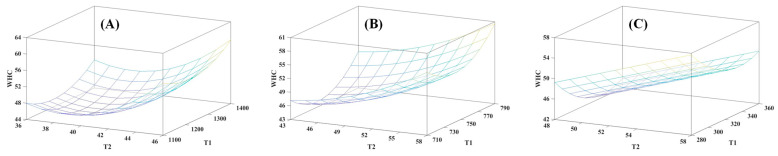
Surface plots of regression models representing longitudinal (T1) and transverse (T2) relaxation times (ms) obtained by magnetic resonance imaging (MRI) and time-domain relaxometry (TD-NMR) comparing water holding capacity (WHC) in the *semitendinosus* muscle. (**A**) MRI 4.7 T; (**B**) MRI 1 T; (**C**) TD-NMR 0.15 T.

Despite obtaining statistically significant models, we hoped to increase the percentage of explanation and the information provided by the models, and for this purpose, longitudinal relaxation time (T1) was included in the equation, obtaining multiple regression models between T1, T2, and WHC (Figure 2 and Table 4). All regression models obtained were statistically significant (*p* < 0.05), and the R^2^ results were 0.92 at 4.7 T, 0.94 at 1 T, and 0.89 at 0.15 T. Therefore, the inclusion of both relaxation times resulted in a substantial increase in R^2^ values compared to those obtained in the linear regression considering only T2, especially at low magnetic fields, where the coefficient of determination obtained indicates that the model would explain nearly 90% of the variability in WHC (compared to 36% explained in the linear regression models).

Obtaining regression models that reveal the WHC, including T1 and T2, in the equation is considered a great advance, since both relaxation times are complementary and are influenced by several aspects (such as mobility and the amount of water, among others). This means that the combination of both parameters provides more complete and detailed information on the sample under study [43].

This inclusion of T1 in the models, as far as the authors have been able to determine, constitutes a novelty in relation to the existing literature, since until now, no regression models including T1 and T2 had been obtained. In this way, the relationship between NMR parameters and WHC is further explored, and more information is provided for the possible application of NMR for the estimation of this parameter. 

As previously mentioned, drip loss is an inverse parameter to WHC and is considered another way of estimating the capacity of the muscle to retain water [5]. For this reason, and using the same procedure that used for WHC, linear regression models with T2 (Table S3) and multiple regression models including T1 (Figure S2 and Table S4) were developed. All the regression models obtained were statistically significant (*p* < 0.05). For the linear regression models, the R^2^ values were 0.60 at 4.7 T, 0.62 at 1 T, and 0.33 at 0.15 T, while for the multiple regression models, the R^2^ increased to 0.88 at 4.7 T, 0.92 at 1 T, and 0.82 at 0.15 T. As expected, the equations of these models exhibited trends opposite to those observed for WHC.

In summary, the combination of T1 and T2 in multiple regression models significantly improved the prediction of WHC and drip loss. These findings highlight the complementary nature of T1 and T2 and demonstrate the potential of NMR-based methodologies for the non-destructive, quantitative assessment of key quality traits in fresh meat.

**Figure 3:**
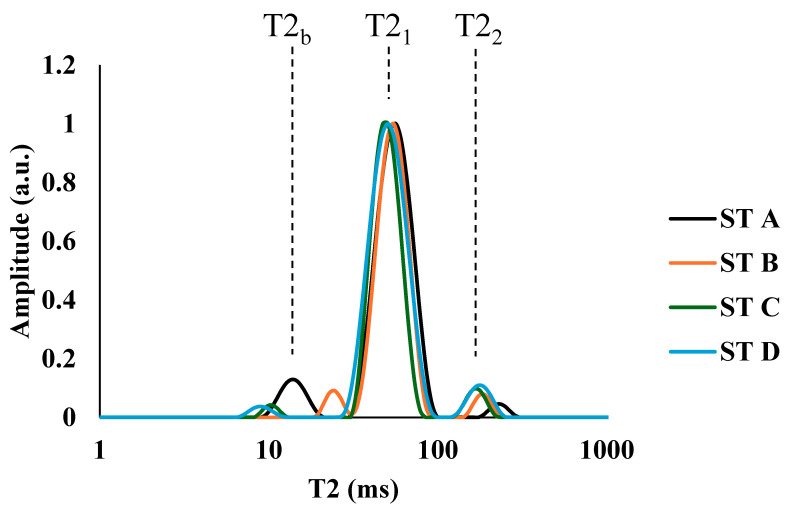
Multiexponential relaxation spectra of transversal relaxation time (T2) obtained at 0.15 T by time-domain nuclear magnetic resonance in *semitendinosus* (ST) muscles of different fat categories (A: ≤10%; B: >10–16%; C: >16–20%; D: >20%).

To further investigate the relationship between WHC and T2, multiexponential analysis was performed using low-field TD-NMR. Average ST muscle relaxation spectra were obtained for each of the fat categories, and the same populations were observed in all of them (Figure 3). A first population was observed around 10 ms, called T2_b_, which was associated with protons from water strongly bound to macromolecules. At around 50 ms, a second population, T2_1_, was observed, which was the majority population in all four fat categories and was associated with the protons of water retained in the three-dimensional network of myofibrillar proteins in the space between the thick and thin filaments. A third population, T22, emerged at approximately 150 ms and was associated with free water. All these populations agreed with those observed by other authors in similar studies on pork [13,14].

**Figure 4:**
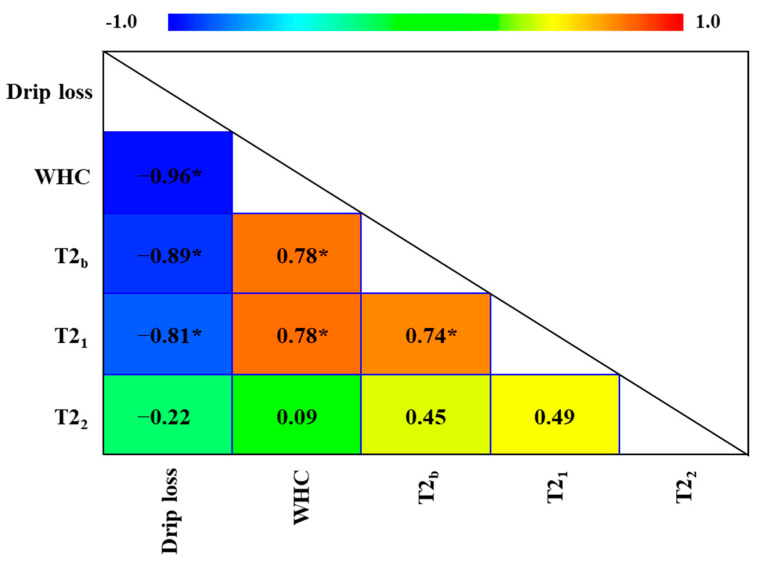
Pearson correlation coefficients of water-holding capacity (WHC), drip loss, and transversal relaxation time (T2) of multiexponential populations (ms) obtained by time-domain relaxometry (0.15 T) in the *semitendinosus* muscle of fresh hams. * *p*-values < 0.05 indicate statistical significance of Pearson’s correlation.

Figure 4 shows the Pearson correlation coefficients between different T2 populations and WHC and drip loss. T2_b_ and T2_1_ exhibited positive correlations with WHC and negative correlations with drip loss. Both water populations are closely related to the protein environment and are characterized by low molecular mobility. The observed correlation patterns are consistent with the behavior of the monoexponential T2 values in relation to WHC and drip loss (Figure 1) because monoexponential T2 reflects a weighted value of multiexponential components, with the dominant population exerting the greatest influence (T2_1_). A positive correlation between WHC and T2_1_ was also reported by other authors in both ST and LD muscles of fresh pork [44,45]. This positive relationship reflects the dependence of WHC on water retention by the protein structure [2]. However, no linear relationship was detected between T2_2_ and either WHC or drip loss. This result may be attributed to the different compartments and dynamic behaviors that free water can exhibit within the muscle structure [2]. Nevertheless, other authors reported negative correlations between T2_2_ and WHC in fresh pork LD muscle [46,47]. The discrepancies among the findings of these studies are attributed to differences in experimental design. Those authors studied selected LD samples based on breed or pH variation [47] or monitored the post-mortem maturation of the LD, which is a predominantly lean muscle [46]. The present study adopts a markedly different approach, focusing on the ST muscle from fresh hams with varying fat levels representative of those commonly used in the meat industry. Huff-Lonergan & Lonergan [2] highlighted the multiple factors influencing WHC, including the intrinsic muscle structure and its distinct compartmentalization, which determines the origin and distribution of drip loss and helps explain the varying behavior among different muscles. In the case of the ST muscle, variations in IMF content, among other factors, may indirectly influence water distribution and mobility. Consequently, these interactions could contribute to the absence of a linear relationship between free water (and consequently, T2_2_) and WHC. In the structure of the ST muscle, particularly in the fatty categories, a part of the free water contributing to the T2_2_ relaxation component may be present in compartments where the distribution of fat, along with the organization of the perimysium and epimysium, prevent its loss through drip, thereby retaining it within the tissue.

From a broader perspective, the results obtained contribute to the current body of knowledge regarding the potential of NMR to provide insights into the structure, composition, and physicochemical properties of different muscles. In particular, the findings underscore the usefulness of NMR for estimating attributes such as WHC and drip loss. In this context, the regression models incorporating T2 and T1 values obtained at high, medium, and low magnetic field strengths demonstrate the robustness, the versatility, and the potential of this methodology not only in laboratory and research environments but also for prospective implementation in meat industry production lines. The determination of WHC by NMR would allow its estimation in an accurate, simple way, without preliminary preparation and with great advantages over traditional methods [5]. Although the integration of NMR equipment into industrial meat processing remains a long-term objective, the findings of this study may serve as a foundation for future research aimed at developing predictive models to ensure the quality of fresh hams, assess their technological suitability, and identify optimal applications in the production of various meat products, as well as for the future development of equipment tailored to industry needs.

## 4. Conclusions

Longitudinal and transverse relaxation times (T1 and T2), derived from MRI and TD-NMR relaxometry using equipment operating at high, medium, and low magnetic field strengths, enabled the development of regression models for the accurate estimation of WHC and drip loss in fresh ham. The ST muscle, in particular, exhibits structural and morphological characteristics that make it suitable for modeling the variability observed in fresh hams with different fat levels within the range typically encountered in the meat industry. This suitability enables the development of correlation models that explain at least 90% of the variability in water retention or loss.

The estimation of WHC and drip loss using MRI and TD-NMR relaxometry offers the advantages of being non-destructive and non-invasive methodologies, and they are also faster and easier to apply compared to the characteristics of conventional techniques.

The results obtained in this study are expected to serve as a foundation for future research aimed at optimizing procedures and establishing NMR as a precise and reliable alternative for the estimation of WHC and drip loss in the meat industry. In contrast to previous studies that relied solely on T2 relaxation times, the inclusion of both T1 and T2 times in the present work enhances the regression models by incorporating more comprehensive structural information. The robust regression models developed from T1 and T2, obtained using equipment operating at high, medium, and low magnetic field strengths, open new avenues for the application of NMR techniques in both analytical laboratories and industrial settings. However, the major limitation of the present study is the adaptation of the models and the standardization of the analyses to the requirements of each industry. Further studies will be required to adapt and refine these techniques to meet the diverse operational requirements of the meat industry. This approach can enable real-time, non-destructive quality control in the production chain.

## Figures and Tables

**Table 1:** (**A**) Chemical composition of *biceps femoris* (BF), *semimembranosus* (SM), and *semitendinosus* (ST) muscles for different fat categories of the fresh hams (A: ≤10%; B: >10–16%; C: >16–20%; D: >20%). DM: dry matter; IMF: intramuscular fat content. (**B**) pH and parameters related to water content of *biceps femoris* (BF), *semimembranosus* (SM), and *semitendinosus* (ST) muscles for different fat categories of the fresh hams (A: ≤10%; B: >10–16%; C: >16–20%; D: >20%). WHC: water holding capacity. (**C**) CIELAB color coordinates of *biceps femoris* (BF), *semimembranosus* (SM), and *semitendinosus* (ST) muscles for different fat categories of the fresh hams (A: ≤10%; B: >10–16%; C: >16–20%; D: >20%).

**(A)**									
**Muscle**	**Fat Category**	**DM (%)**		**Ash (DM) (%)**		**IMF (DM) (%)**		**Protein (DM) (%)**	
BF	A	25.88 ± 0.73	b, γ	4.18 ± 0.14	a, α	14.05 ± 0.81	b, γ	81.28 ± 1.02	b, α
SM	A	26.01 ± 0.34	b, βγ	4.49 ± 0.09	a, β	11.13 ± 0.54	c, γ	83.92 ± 0.42	a, α
ST	A	27.23 ± 0.67	a, β	4.11 ± 0.52	a, αβ	21.69 ± 0.89	a, β	73.62 ± 0.78	c, α
BF	B	26.75 ± 0.67	a, βγ	3.73 ± 0.34	a, β	16.11 ± 1.76	b, β	79.75 ± 1.70	b, β
SM	B	25.52 ± 0.30	b, γ	4.12 ± 0.30	a, γ	13.32 ± 0.68	c, β	82.10 ± 0.48	a, β
ST	B	26.35 ± 0.25	a, γ	3.73 ± 0.38	a, β	20.87 ± 0.82	a, β	74.90 ± 0.78	c, α
BF	C	28.31 ± 0.69	a, α	4.04 ± 0.44	b, αβ	21.34 ± 1.16	b, α	74.32 ± 0.60	b, δ
SM	C	26.19 ± 0.33	b, β	4.79 ± 0.21	a, α	11.65 ± 1.70	c, βγ	83.16 ± 1.41	a, αβ
ST	C	28.10 ± 0.16	a, α	4.40 ± 0.34	ab, α	23.53 ± 1.05	a, α	71.62 ± 1.15	c, β
BF	D	27.09 ± 0.51	b, β	3.96 ± 0.32	b, αβ	20.05 ± 0.49	b, α	75.81 ± 0.52	a, γ
SM	D	27.29 ± 0.48	b, α	4.40 ± 0.08	a, β	18.62 ± 2.13	b, α	76.51 ± 2.22	a, γ
ST	D	28.46 ± 0.36	a, α	4.15 ± 0.06	ab, αβ	23.89 ± 1.23	a, α	71.50 ± 1.06	b, β
(**B**)									
**Muscle**	**Fat Category**	**pH**		**WHC (%)**		**Drip Loss (%)**			
BF	A	5.77 ± 0.05	ab, β	48.44 ± 1.78	a, αβ	4.10 ± 1.03		ab, αβ	
SM	A	5.72 ± 0.09	b, α	48.18 ± 2.31	a, αβ	5.07 ± 0.84		a, βγ	
ST	A	5.88 ± 0.12	a, α	50.11 ± 0.83	a, αβ	3.44 ± 0.72		b, βγ	
BF	B	5.94 ± 0.34	a, αβ	50.38 ± 1.42	ab, α	3.46 ± 0.49		b, β	
SM	B	5.76 ± 0.22	a, α	48.71 ± 0.77	b, α	5.04 ± 0.54		a, γ	
ST	B	5.88 ± 0.46	a, α	51.14 ± 1.38	a, α	3.19 ± 0.61		b, γ	
BF	C	6.13 ± 0.25	a, α	46.91 ± 2.48	a, β	5.04 ± 1.30		a, α	
SM	C	5.87 ± 0.17	a, α	46.35 ± 1.49	a, β	6.19 ± 0.78		a, α	
ST	C	6.02 ± 0.27	a, α	46.84 ± 2.95	a, γ	4.73 ± 1.30		a, α	
BF	D	6.10 ± 0.09	a, α	48.10 ± 2.48	a, αβ	4.74 ± 1.08		b, αβ	
SM	D	5.92 ± 0.07	b, α	46.91 ± 1.18	a, αβ	5.97 ± 0.48		a, αβ	
ST	D	6.11 ± 0.14	a, α	47.90 ± 1.42	a, βγ	4.56 ± 0.54		b, αβ	
(**C**)									
**Muscle**	**Fat Category**	L*		a*		b*			
BF	A	51.13 ± 1.67	a, α	9.65 ± 0.79	b, β	4.96 ± 0.65		b, α	
SM	A	53.66 ± 2.40	a, α	8.15 ± 0.93	c, β	5.76 ± 0.81		b, α	
ST	A	51.67 ± 1.64	a, α	11.66 ± 1.30	a, γ	6.74 ± 0.38		a, αβ	
BF	B	48.32 ± 0.48	a, β	11.85 ± 0.71	a, α	5.50 ± 0.40		ab, α	
SM	B	50.61 ± 1.34	a, β	8.96 ± 0.53	b, β	4.72 ± 0.07		b, β	
ST	B	48.73 ± 2.54	a, β	12.76 ± 1.61	a, βγ	6.45 ± 1.22		a, β	
BF	C	46.95 ± 1.91	b, β	12.26 ± 0.63	b, α	5.07 ± 0.54		b, α	
SM	C	46.78 ± 1.19	b, γ	10.57 ± 1.17	c, α	5.06 ± 0.26		b, αβ	
ST	C	51.25 ± 1.94	a, αβ	14.75 ± 0.87	a, α	8.04 ± 0.97		a, α	
BF	D	46.82 ± 0.85	b, β	12.25 ± 0.83	a, α	5.21 ± 0.22		b, α	
SM	D	48.96 ± 1.84	a, βγ	10.70 ± 0.55	b, α	5.45 ± 0.69		b, αβ	
ST	D	54.14 ± 5.85	a, αβ	12.18 ± 1.55	a, αβ	7.13 ± 1.28		a, αβ	

a, b, c: values with different letters corresponding to different muscles of the same fat content category differ significantly (*p* < 0.05). α, β, γ, δ: values with different letters corresponding to the same muscle of different fat content categories differ significantly (*p* < 0.05).

**Table 2:** Multifactorial analysis of the different physicochemical parameters of fresh hams as a function of muscle type [*biceps femoris* (BF), *semimembranosus* (SM), and *semitendinosus* (ST)] and fat category.

Parameters	Factors					
	Muscle (M)		Fat Category (F)		MxF	
	*F*-Value	*p*-Value	*F*-Value	*p*-Value	*F*-Value	*p*-Value
**Chemical composition (%)**						
Dry matter (DM)	34.01	<0.0001	34.07	<0.0001	8.20	<0.0001
Ash content (DM)	12.81	<0.0001	8.66	0.0001	0.70	0.6509
Protein content (DM)	281.56	<0.0001	61.85	<0.0001	16.62	<0.0001
Intramuscular fat content (DM)	265.01	<0.0001	54.55	<0.0001	15.86	<0.0001
**Physicochemical characteristics**						
pH	3.46	0.0395	4.27	0.0094	0.28	0.9431
**Water parameters**						
Water holding capacity (WHC) (%)	3.24	0.0479	9.73	<0.0001	0.48	0.8216
Drip loss (%)	18.72	<0.0001	9.52	<0.0001	0.18	0.9823
**Color parameters**						
L*	9.19	0.0004	15.35	<0.0001	4.33	0.0014
a*	63.18	<0.0001	21.53	<0.0001	1.01	0.4327
b*	44.4	<0.0001	1.27	0.2961	2.85	0.0185

*p*-values < 0.05 indicate significant interaction of the factor with the studied parameter.

**Table 3:** Longitudinal (T1) and transverse (T2) relaxation times (ms) obtained by magnetic resonance imaging (MRI) (1 and 4.7 T) and time-domain relaxometry (TD-NMR) (0.15 T) of *semitendinosus* (ST) muscle for different fat categories of the fresh hams (A: ≤10%; B: >10–16%; C: >16–20%; D: >20%).

Fat	T2 (4.7 T)		T1 (4.7 T)		T2 (1 T)		T1 (1 T)		T2 (0.15 T)		T1 (0.15 T)	
A	43.50 ± 0.23	a	1312.20 ± 15.75	a	51.85 ± 1.06	a	739.47 ± 29.06	b	56.91 ± 0.86	a	337.55 ± 1.44	b
B	43.84 ± 1.14	a	1308.40 ± 44.83	a	52.70 ± 2.47	a	767.59 ± 5.91	a	54.67 ± 0.10	b	370.86 ± 0.64	a
C	40.65 ± 2.61	b	1265.40 ± 51.26	a	47.28 ± 3.57	b	752.78 ± 18.26	ab	50.02 ± 1.50	d	306.86 ± 18.08	c
D	42.96 ± 0.44	a	1266.00 ± 24.85	a	51.54 ± 1.39	a	741.97 ± 8.97	b	52.82 ± 1.08	c	346.04 ± 0.67	b

a, b, c, d: values with different letters corresponding to the same muscle of different fat content categories differ significantly (*p* < 0.05).

**Table 4:** Regression model equations and statistical parameters of water holding capacity (WHC) and longitudinal (T1) and transverse relaxation times (T2) obtained by magnetic resonance imaging (1 T and 4.7 T) and time-domain relaxometry (0.15 T) in the *semitendinosus* muscle. The R^2^, adjusted R^2^, standard error (SE), and *p*-value are indicated for each model.

Dependent Variable	Independent Variable	Regression Coefficient	SE	F-Value	*p*-Value	R^2^ Model
WHC	constant	567.415	129.249			0.94
	T2 (4.7 T) (ms)	−15.4179	3.17987	23.51	0.0002	**Adj. R^2^ model**
	T1 (4.7 T) (ms)	−0.367942	0.172053	4.57	0.0493	0.92
	T2^2^ (4.7 T) (ms)	0.198722	0.0383775	26.81	0.0001	**SE model**
	T1^2^ (4.7 T) (ms)	0.00015104	0.0000663681	5.18	0.0380	0.69
						***p*-value model**
						<0.0001
						**F-value model**
						55.10
**Dependent Variable**	**Independent Variable**	**Regression Coefficient**	**SE**	**F-Value**	***p*-Value**	**R^2^ Model**
WHC	constant	1719.87	223.446			0.95
	T2 (1 T) (ms)	−2.63615	1.13718	5.37	0.0350	**Adj. R^2^ model**
	T1 (1 T) (ms)	−4.3731	0.571921	58.47	<0.0001	0.94
	T2^2^ (1 T) (ms)	0.0324571	0.0113611	8.16	0.0120	**SE model**
	T1^2^ (1 T) (ms)	0.00294693	0.000383083	59.18	<0.0001	0.60
						***p*-value model**
						<0.0001
						**F-value model**
						73.28
**Dependent Variable**	**Independent Variable**	**Regression Coefficient**	**SE**	**F-Value**	***p*-Value**	**R^2^ Model**
WHC	constant	242.059	27.6622			0.91
	T2 (0.15 T) (ms)	0.846445	0.0895625	89.32	<0.0001	**Adj. R^2^ model**
	T1 (0.15 T) (ms)	−1.42949	0.175391	66.43	<0.0001	0.89
	T1^2^ (0.15 T) (ms)	0.00212762	0.000261601	66.15	<0.0001	**SE model**
						0.79
						***p*-value model**
						<0.0001
						**F-value model**
						42.97

*p*-values < 0.05 indicate statistical significance of the regression models and of each parameter.

## Data Availability

The original contributions presented in the study are included in the article/Supplementary Material, further inquiries can be directed to the corresponding author.

## Supplementary Materials

The following supporting information can be downloaded at: https://www.mdpi.com/article/10.3390/foods14132329/s1, Table S1: Description of the fresh ham characteristics considering the fat content classification; Figure S1: Cross-section of fresh deboned pork leg including the *biceps femoris* (BF), *semimembranosus* (SM), and *semitendinosus* (ST) muscles; Table S2: Regression model equations of water holding capacity (WHC) and transverse relaxation time (T2) obtained by magnetic resonance imaging (1 T and 4.7 T) and time-domain relaxometry (0.15 T) in the *semitendinosus* muscle; Table S3: Regression model equations of drip loss and transverse relaxation time (T2) obtained by magnetic resonance imaging (1 T and 4.7 T) and time-domain relaxometry (0.15 T) in the *semitendinosus* muscle; Figure S2: Surface plots of regression models representing longitudinal (T1) and transverse (T2) relaxation times (ms) obtained by magnetic resonance imaging (MRI) and time-domain relaxometry (TD-NMR) against drip loss (%) in the *semitendinosus* muscle. (A) MRI 4.7 T; (B) MRI 1 T; (C) TD-NMR 0.15 T; Table S4: Regression models equations and statistical parameters of drip loss and longitudinal (T1) and transverse relaxation times (T2) obtained by magnetic resonance imaging (1 T and 4.7 T) and time-domain relaxometry (0.15 T) in the *semitendinosus* muscle.

## References

[1] Tornberg E.V.A. (2005). Effects of heat on meat proteins–Implications on structure and quality of meat products. Meat Sci..

[2] Huff-Lonergan E., Lonergan S.M. (2005). Mechanisms of water-holding capacity of meat: The role of postmortem biochemical and structural changes. Meat Sci..

[3] Warner R.D. (2023). The eating quality of meat: IV—Water holding capacity and juiciness. Lawrie’s Meat Science.

[4] Barbut S. (2024). Measuring water holding capacity in poultry meat. Poult. Sci..

[5] Oswell N.J., Gilstrap O.P., Pegg R.B. (2021). Variation in the terminology and methodologies applied to the analysis of water holding capacity in meat research. Meat Sci..

[6] Günther H. (2013). NMR Spectroscopy: Basic Principles, Concepts and Applications in Chemistry.

[7] Keeler J. (2011). Understanding NMR Spectroscopy.

[8] Liang Z.P., Lauterbur P.C. (1999). Principles of Magnetic Resonance Imaging: A Signal Processing Perspective.

[9] García-García A.B., Fernández-Valle M.E., Castejón D., Escudero R., Cambero M.I. (2019). Use of MRI as a predictive tool for physicochemical and rheological features during cured ham manufacturing. Meat Sci..

[10] Bertram H., Andersen H. (2006). Proton NMR Relaxometry in Meat Science. Modern Magnetic Resonance.

[11] García-García A.B., Cambero M.I., Castejón D., Escudero R., Fernández-Valle M.E. (2019). Dry cured-ham microestructure: A T2 NMR relaxometry, SEM and uniaxial tensile test combined study. Food Struct..

[12] Khan M.A., Ahmad B., Kamboh A.A., Qadeer Z. (2022). Use of NMR relaxometry for determination of meat properties: A brief review. Food Mater. Res..

[13] Remiro V., Cambero M.I., Romero-de-Ávila M.D., Castejón D., Moreno-Molera D., Segura J., Fernández-Valle M.E. (2025). Monitoring salting kinetics of pork loin using magnetic resonance imaging (MRI) and time-domain nuclear magnetic resonance (TD-NMR). LWT.

[14] Cônsolo N.R., de Paula A.P., Rezende-de-Souza J.H., Herreira V.L., Gôngora A.L.S., Colnago L.A., Moraes T.B., Santos P.M., Nassu R.T., Pflanzer S.B. (2024). Assessment of water relaxometry of meat under different ageing processes using time domain nuclear magnetic resonance relaxometry. Food Res. Int..

[15] Guo Z., Chen C., Ma G., Yu Q., Zhang L. (2023). LF-NMR determination of water distribution and its relationship with protein- related properties of yak and cattle during postmortem aging. Food Chem. X.

[16] Bianchi M., Capozzi F., Cremonini M.A., Laghi L., Petracci M., Placucci G., Cavani C. (2004). Influence of the season on the relationships between NMR transverse relaxation data and water-holding capacity of turkey breast meat. J. Sci. Food Agric..

[17] Hu H.H., Nayak K.S. (2010). Change in the proton T1 of fat and water in mixture. Magn. Reson. Med..

[18] AOAC (1996). Official method 991.36. Fat (crude) in meat and meat products. Soxhlet extraction method. Official Methods of Analysis.

[19] AOAC (2006). Official method 950.46. Moisture in Meat. Official Methods of Analysis.

[20] Kauffman R.G., Eikelenboom G., Van der Wal P.G., Engel B., Zaar M. (1986). A comparison of methods to estimate water-holding capacity in post-rigor porcine muscle. Meat Sci..

[21] AOAC (2012). Official method 928.08. Protein in meat. Kjeldahl method. Official Methods of Analysis.

[22] Bjarnason T.A., Mitchell J.R. (2010). AnalyzeNNLS: Magnetic resonance multiexponential decay image analysis. J. Magn. Reson..

[23] Chatterjee S., Hadi A.S. (2015). Variable selection procedures. Regression Analysis by Example.

[24] Ventanas J. (2012). Jamón Ibérico y Serrano. Fundamentos de la Elaboración y de la Calidad.

[25] Lebret B., Čandek-Potokar M. (2022). Pork quality attributes from farm to fork. Part II. Processed pork products. Animal.

[26] Cernadas E., Fernández-Delgado M., Fulladosa E., Muñoz I. (2022). Automatic marbling prediction of sliced dry-cured ham using image segmentation, texture analysis and regression. Expert. Syst. Appl..

[27] Romero de Ávila M.D., Escudero R., Ordóñez J.A., Cambero M.I. (2014). Weibull analysis characterizes the breaking properties of dry-cured ham slices. Meat Sci..

[28] Kim J.H., Seong P.N., Cho S.H., Park B.Y., Hah K.H., Yu L.H., Lim D.G., Hwang I.H., Kim D.H., Lee J.M. (2008). Characterization of nutritional value for twenty-one pork muscles. Asian-Australas. J. Anim. Sci..

[29] Font-i-Furnols M., Brun A., Gispert M. (2019). Intramuscular fat content in different muscles, locations, weights and genotype-sexes and its prediction in live pigs with computed tomography. Animal.

[30] Ruusunen M., Puolanne E. (2004). Histochemical properties of fibre types in muscles of wild and domestic pigs and the effect of growth rate on muscle fibre properties. Meat Sci..

[31] Realini C.E., Vénien A., Gou P., Gatellier P., Pérez-Juan M., Danon J., Astruc T. (2013). Characterization of Longissimus thoracis, Semitendinosus and Masseter muscles and relationships with technological quality in pigs. 1. Microscopic analysis of muscles. Meat Sci..

[32] Hwang Y.H., Kim G.D., Jeong J.Y., Hur S.J., Joo S.T. (2010). The relationship between muscle fiber characteristics and meat quality traits of highly marbled Hanwoo (Korean native cattle) steers. Meat Sci..

[33] Lebedová N., Stupka R., Čítek J., Zadinová K., Kudrnáčová E., Okrouhlá M., Dundáčková P. (2019). Muscle fibre types and their relation to meat quality traits in pigs. Sci. Agric. Bohem..

[34] Watanabe G., Motoyama M., Nakajima I., Sasaki K. (2017). Relationship between water-holding capacity and intramuscular fat content in Japanese commercial pork loin. Asian-Australas. J. Anim. Sci..

[35] Daszkiewicz T., Bąk T., Denaburski J. (2005). Quality of pork with a different intramuscular fat (IMF) content. Pol. J. Food Nutr. Sci..

[36] Graziotti G.H., Rodríguez Menéndez J., Ríos M.C., Salinas M., Paltenghi Ceschel A., Affricano O., Bosco A., Victorica C., Basso L. (2007). Perfil metabólico del músculo semitendinoso del cerdo. InVet.

[37] Semenova A.A., Pchelkina V.A., Nasonova V.V., Loskutov S.I., Bogolyubova N.V., Nekrasov R.V., Motovilina A.A., Bogdanova Y.I. (2023). Histological characteristics and functional properties of red and white parts of m. semitendinosus of slaughter pigs. Theory Pract. Meat Process..

[38] Stanisz G.J., Odrobina E.E., Pun J., Escaravage M., Graham S.J., Bronskill M.J., Henkelman R.M. (2005). T1, T2 relaxation and magnetization transfer in tissue at 3T. Magn. Reson. Med..

[39] Marty B., Carlier P.G. (2019). Physiological and pathological skeletal muscle T1 changes quantified using a fast inversion-recovery radial NMR imaging sequence. Sci. Rep..

[40] Jo K., Lee S., Jeong H.G., Lee D.H., Yoon S., Chung Y., Jung S. (2023). Utilization of electrical conductivity to improve prediction accuracy of cooking loss of pork loin. Food Sci. Anim. Resour..

[41] Szmańko T., Lesiów T., Górecka J. (2021). The water-holding capacity of meat: A reference analytical method. Food Chem..

[42] Bertram H.C., Andersen H.J. (2007). NMR and the water-holding issue of pork. J. Anim. Breed. Genet..

[43] Mariette F. (2009). Investigations of food colloids by NMR and MRI. Curr. Opin. Colloid Interface Sci..

[44] Bertram H.C., Andersen H.J., Karlsson A.H. (2001). Comparative study of low-field NMR relaxation measurements and two traditional methods in the determination of water holding capacity of pork. Meat Sci..

[45] Brøndum J., Munck L., Henckel P., Karlsson A., Tornberg E., Engelsen S.B. (2000). Prediction of water-holding capacity and composition of porcine meat by comparative spectroscopy. Meat Sci..

[46] Zhu H., O’Farrell M., Bouquet G., Lunde K., Egelandsdal B., Alvseike O., Berg P., Gjerlaug-Enger E., Hansen E.W. (2016). Evaluating nuclear magnetic resonance (NMR) as a robust reference method for online spectroscopic measurement of water holding capacity (WHC). J. Food Eng..

[47] Straadt I.K., Rasmussen M., Andersen H.J., Bertram H.C. (2007). Aging-induced changes in microstructure and water distribution in fresh and cooked pork in relation to water-holding capacity and cooking loss–A combined confocal laser scanning microscopy (CLSM) and low-field nuclear magnetic resonance relaxation study. Meat Sci..

