# Supplementary material for: Contributions to Estimating the Water-Holding Capacity in Fresh Pork Hams Using NMR Relaxometry
Source: Foods. 2025 Jun 30;14(13):2329. doi: 10.3390/foods14132329 (PMC12248548; doi:10.3390/foods14132329)
Supplement: Supplementary file 1 [file foods-14-02329-s001.zip › foods-3705173-supplementary.pdf]

Table S1: Description of the fresh hams characteristics considering the fat content classification.

Fat category	Total fat range (%)	Total fat content (%)	pH	Fresh ham weight (kg)
A	≤ 10	8.11 ± 1.12	5.65 ± 0.04	12.35 ± 0.15
B	> 10-16	12.45 ± 0.81	5.63 ± 0.05	12.48 ± 0.17
C	> 16-20	17.96 ± 0.25	5.67 ± 0.06	12.64 ± 0.28
D	> 20	24.98 ± 2.10	5.65 ± 0.06	12.45 ± 0.24

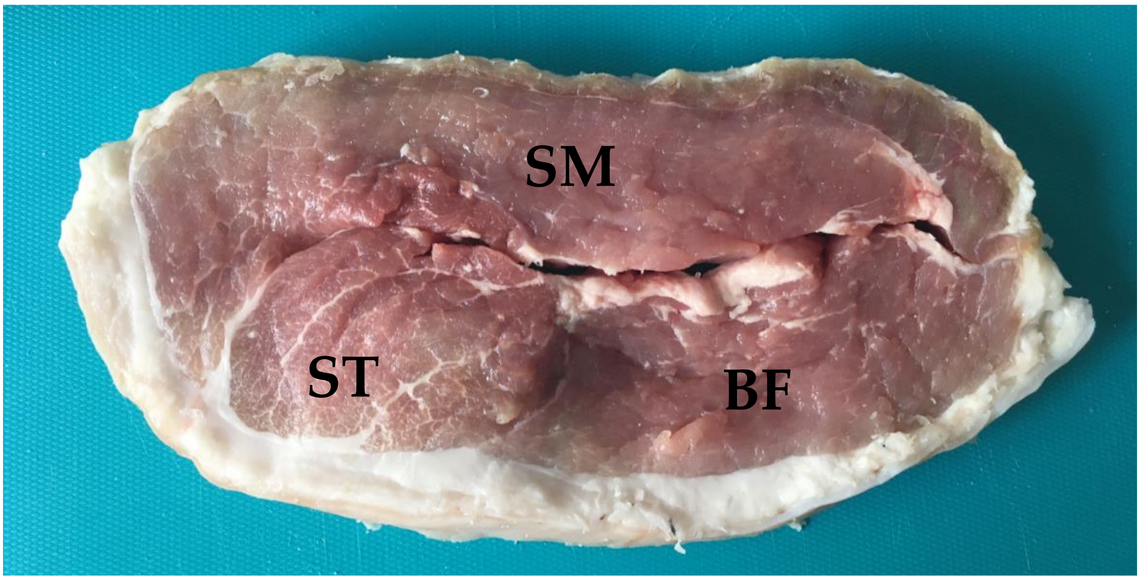

Figure S1: Cross section of fresh deboned pork leg including the *biceps femoris* (BF), *semimembranosus* (SM) and *semitendinosus* (ST) muscles.

Table S2: Regression model equations of water holding capacity (WHC) and transverse relaxation time (T2) obtained by magnetic resonance imaging (1 T and 4.7 T) and time-domain relaxometry (0.15 T) in the *semitendinosus* muscle. The R^2, adjusted R^2, standard error (SE) and *p*-value are indicated for each model. *p*-values < 0.05 indicate statistical significance of the regression models.

Dependent Variable	Independent Variable	Regression coefficient	SE	*p*-value	R^2 model
WHC	constant	1.42304	7.10145	0.8434	0.71
	T2 (4.7 T) (ms)	1.11314	0.166015	< 0.0001	**Adj. R^2 model**
					0.70
					**SE model**
					1.33
					**RMSE model**
					0.22
					***p*-value model**
					< 0.0001
					**F-value model**
					44.96

Dependent Variable	Independent Variable	Regression coefficient	SE	*p*-value	R^2 model
WHC	constant	14.7683	5.13522	0.0101	0.71
	T2 (1 T) (ms)	0.67324	0.100834	< 0.0001	**Adj. R^2 model**
					0.70
					**SE model**
					1.34
					**RMSE model**
					0.26
					***p*-value model**
					< 0.0001
					**F-value model**
					44.58

Dependent Variable	Independent Variable	Regression coefficient	SE	*p*-value	R^2 model
WHC	constant	19.1917	8.65257	0.0397	0.40
	T2 (0.15 T) (ms)	0.556016	0.161214	0.0029	**Adj. R^2 model**
					0.36
					**SE model**
					1.93
					**RMSE model**
					0.33
					***p*-value model**
					0.0029
					**F-value model**
					11.90

Table S3: Regression model equations of drip loss and transverse relaxation time (T2) obtained by magnetic resonance imaging (1 T and 4.7 T) and time-domain relaxometry (0.15 T) in the *semitendinosus* muscle. The R^2, adjusted R^2, standard error (SE) and *p*-value are indicated for each model. *p*-values < 0.05 indicate statistical significance of the regression models.

**Dependent Variable**	**Independent Variable**	**Regression coefficient**	**SE**	***p*-value**	**R^2 model**
Drip loss	constant	22.9586	3.52737	< 0.0001	0.62
	T2 (4.7 T) (ms)	-0.444068	0.0824614	< 0.0001	**Adj. R^2 model**
					0.60
					**SE model**
					0.66
					**RMSE model**
					0.12
					***p*-value model**
					< 0.0001
					**F-value model**
					29.00

**Dependent Variable**	**Independent Variable**	**Regression coefficient**	**SE**	***p*-value**	**R^2 model**
Drip loss	constant	17.9204	2.46025	< 0.0001	0.64
	T2 (1 T) (ms)	-0.274198	0.048309	< 0.0001	**Adj. R^2 model**
					0.62
					**SE model**
					0.64
					**RMSE model**
					0.12
					***p*-value model**
					< 0.0001
					**F-value model**
					32.22

**Dependent Variable**	**Independent Variable**	**Regression coefficient**	**SE**	***p*-value**	**R^2 model**
Drip loss	constant	16.1788	3.82285	0.0005	0.36
	T2 (0.15 T) (ms)	-0.227572	0.0712268	0.0050	**Adj. R^2 model**
					0.33
					**SE model**
					0.85
					**RMSE model**
					0.15
					***p*-value model**
					0.0050
					**F-value model**
					10.21

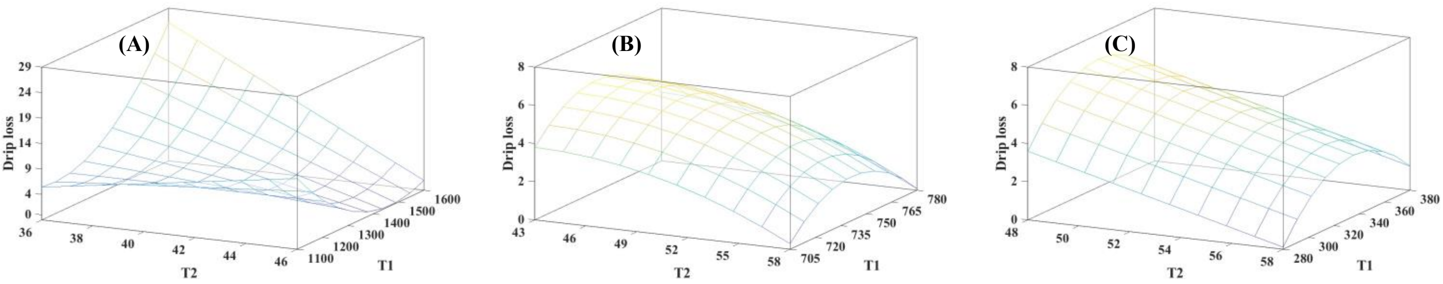

Figure S2: Surface plots of regression models representing longitudinal (T1) and transverse (T2) relaxation times (ms) obtained by magnetic resonance imaging (MRI) and time-domain relaxometry (TD-NMR) against drip loss (%) in the *semitendinosus* muscle. (A) MRI 4.7 T; (B) MRI 1 T; (C) TD-NMR 0.15 T.

Table S4: Regression models equations and statistical parameters of drip loss and longitudinal (T1) and transverse relaxation times (T2) obtained by magnetic resonance imaging (1 T and 4.7 T) and time-domain relaxometry (0.15 T) in the *semitendinosus* muscle. The R^2, adjusted R^2, standard error (SE) and *p*-value are indicated for each model. *p*-values < 0.05 indicate statistical significance of the regression models and of each parameter.

Dependent Variable	Independent Variable	Regression coefficient	SE	F-Value	P-Value	R^2 model
	constant	-145.374	38.1935			0.90
	T2 (4.7 T) (ms)	7.93277	1.85896	18.21	0.0009	**Adj. R^2 model**
Drip loss	*$T1^2$ (4.7 T) (ms)*	0.000102474	0.0000235831	18.88	0.0008	0.88
	T2 × T1 (4.7 T) (ms)	-0.00653451	0.0014603	20.02	0.0006	**SE model**
						0.38
						***p*-value model**
						< 0.0001
						**F-value model**
						38.78
**Dependent Variable**	**Independent Variable**	**Regression coefficient**	**SE**	**F-Value**	**P-Value**	**R^2 model**
	constant	-852.18	112.175			0.94
	T2 (1 T) (ms)	1.27097	0.54255	5.49	0.0357	**Adj. R^2 model**
Drip loss	*T1 (1 T) (ms)*	2.2409	0.28947	59.93	< 0.0001	0.92
	$T2^2$ (1 T) (ms)	-0.0149051	0.00542143	7.56	0.0166	**SE model**
	$T1^2$ (1 T) (ms)	-0.00151088	0.000194211	60.52	< 0.0001	0.29
						***p*-value model**
						< 0.0001
						**F-value model**
						53.06
**Dependent Variable**	**Independent Variable**	**Regression coefficient**	**SE**	**F-Value**	**P-Value**	**R^2 model**
	constant	-89.3499	15.4028			0.86
	T2 (0.15 T) (ms)	-0.348742	0.0532704	42.86	< 0.0001	**Adj. R^2 model**
Drip loss	*T1 (0.15 T) (ms)*	0.671899	0.097146	47.84	< 0.0001	0.82
	$T1^2$ (0.15 T) (ms)	-0.00100059	0.000144495	47.95	< 0.0001	**SE model**
						0.45
						***p*-value model**
						< 0.0001
						**F-value model**
						25.99
